# Pim2 is important for regulating DNA damage response in multiple myeloma cells

**DOI:** 10.1038/bcj.2016.73

**Published:** 2016-08-26

**Authors:** J Ramachandran, L Santo, K T Siu, C Panaroni, N Raje

**Affiliations:** 1 Massachusetts General Hospital Cancer Center, MGH Cancer Center, Harvard Medical School, Boston, MA, USA

## Abstract

Pan proviral integrations of Moloney virus (PIM) inhibition in multiple myeloma (MM) results in reduced cell viability in tested human-derived MM cell lines and reduces tumor burden in xenograft mouse models, making PIMs important therapeutic targets for the disease. PIM kinase inhibitors are currently being tested clinically in MM. We sought to elucidate the role of the various PIMs in MM. Our data demonstrate that Pim2 has a significant role in MM cell cytotoxicity. Our data provide evidence for a novel role for Pim2 in the regulation of the DNA damage response (DDR). Knockdown of Pim2 upregulates several downstream DDR markers, mimicking the effects of doxorubicin (Dox) treatment of MM cells, and suggesting a role for the kinase as a negative regulator of this pathway. Dox-induced DNA damage results in a decrease in Pim2 levels, placing the kinase directly downstream of the site of Dox-DNA binding. Overexpression of Pim2 confers a slight survival advantage against Dox through antiapoptotic activity, further underscoring its relevance in the DDR pathway. These data provide insights into a novel mechanism of PIM kinase activity and provide the framework for designing therapeutic approaches in MM.

## Introduction

The proviral integrations of Moloney virus (PIM) kinases are serine-threonine kinases that have recently been shown to have a multitudinous and integral role in the evolution and progression of many hematological malignancies.^1, 2^ In multiple myeloma (MM) they occupy an important stratum of kinases that promote cancer cell proliferation and protect from apoptosis.^3^ The PIM kinase family is composed of three serine-threonine kinase isoforms; PIM1, 2 and 3, which are constitutively active in cancer cells.^4^ Translation of the PIM kinases is promoted by cytokine-mediated activation of the JAK-STAT (Janus kinase/signal transducers and activators of transcription) and NF-κB (nuclear factor-κB) pathways, thus causing an increase in PIM expression levels in MM cells when in coculture with the bone marrow stromal cell (BMSC) compartment.^3^ Interleukin-6 (IL-6) is secreted by BMSCs into the microenvironment and activates the STAT3 pathway in MM cells to promote PIM transcription.^3^ In MM cells, the PIMs act as prosurvival factors to phosphorylate Bcl-2-associated agonist of cell death (BAD) and prevent apoptosis.^5^ PIM2 further promotes cell proliferation by phosphorylating the active suppressant of mammalian target of rapamycin complex 1 activity, TSC2, and causing it to dissociate with mammalian target of rapamycin complex 1.^6^ PIM inhibition results in a decrease in phosphorylated 4EBP1 (eukaryotic translation initiation factor 4E-binding protein 1) as well as a decrease in MCL1 and c-MYC levels.^3^ PIM inhibition has demonstrated a role for the kinases in cell cycle arrest as well as apoptosis in cell culture, whereas reduced MM tumor burden has been observed in a xenograft mouse model.^7^ Despite growing interest in these kinases as therapeutic molecular targets, there is a surprising shortage of effective small-molecule inhibitors in the clinic for MM treatment. Inhibitors that have previously made it to clinical trials for MM have been pan-PIM inhibitors with varying degrees of efficacy in targeting each isoform.^4, 8^ In this study, we sought to elucidate the differential roles of each of the PIM isoforms, and in so doing, gain a better understanding of what mode of targeting would be most relevant to the treatment of MM.

## Materials and methods

### Reagents

Bortezomib and doxorubicin (Dox) were purchased from Selleck Chemicals LLC (Houston, TX, USA).

### MM cell lines

The MM cell lines U266 and RPMI-8226 were purchased from American Type Culture Collection (Rockville, MD, USA). Dr Steven Rosen (Northwestern University, Chicago, IL, USA) provided dexamethasone-sensitive (MM1.S) and dexamethasone-resistant (MM1.R) human cell lines. Melphalan-resistant (LR5) and Dox-resistant RPMI-Dox40 (Dox40) cell lines were provided by Dr William Dalton (H Lee Moffitt Cancer Center, Tampa, FL, USA). OPM1 and OPM2 cells were obtained from Dr P Lief Bergsagel (Mayo Clinic, Scottsdale, AZ, USA). ANBL6 WT and ANBL6 velcade-resistant (ANBL6-VR) cells were provided by Dr Robert Orlowski (MD Anderson Cancer Center, Houston, TX, USA) and INA-6 cells were provided by Dr Renate Burger (University of Kiel, Keil, Germany). The cell lines were cultured in RPMI-1640 medium containing 10% fetal bovine serum (Gibco, Life Technologies, Carlsbad, CA, USA), 2 μm l-glutamine, 100 U/ml penicillin and 100 μg/ml streptomycin (Gibco). The ANBL6 cells were cultured in 20% fetal bovine serum with 2.5 ng/ml of IL-6 (the VR line was cultured with 1 nm/ml of bortezomib). The INA-6 cells were also cultured in 2.5 ng/ml of IL-6 (R&D Systems, Minneapolis, MN, USA).

### Mononuclear cell separation and processing of patient samples

Bone marrow samples collected from MM patients in various stages of disease were processed by Ficoll-Paque (GE Healthcare, Boston, MA, USA) gradient to obtain mononuclear cells. These cells were then sorted into CD138+ and CD138− fractions by magnetic bead separation (MACS Separation Columns; Miltenyi Biotec, Cambridge, MA, USA) and stored as dry frozen pellets for further analysis. A portion of the negative fraction of cells were also plated and cultured in α-MEM culture media with 20% fetal bovine serum, 2 μm/l  l-glutamine, 100 U/ml penicillin and 100 μg/ml streptomycin (Gibco, Life Technologies). These cells were kept in culture for up to 2 months and was used as the BMSC compartment for *in vitro* coculture studies. Informed consent was obtained in accordance with the Declaration of Helsinki, under the approval of the Institutional Review Board of the Massachusetts General Hospital, for all patient samples.

### Western blotting

MM cells were harvested and lysed as mentioned previously.^9^ Lysates were then measured for protein quantification, boiled (100 °C for 5 min), run on sodium dodecyl sulfate-polyacrylamide gel electrophoresis 4–15% gradient gels at 110 V (Bio-Rad, Hercules, CA, USA), transferred to nitrocellulose membranes and immunoblotted with antibodies to the following: pCHK2 (Thr68), pCHK1 (Ser345), pH2AX (Ser139), PIM1, PIM2, PIM3, Caspase-3, PARP, BAD, pBAD (Ser112), p21 Waf1/Cip1, MDR-1, pYAP1 (Ser127), YAP1, ABL1 and GAPDH (Cell Signaling, Beverly, MA, USA), which was used as a loading control. All antibodies were diluted 1:1000 in 5% bovine serum albumin/TBST (TBS plus Tween-20).

### Cell viability assays

The effects of the pan-PIM kinase inhibitor AZD1208 and Dox, and that of the single kinase knockdowns and knock-ins on the survival of MM cell lines was analyzed by measuring MTT (3-(4,5-dimethylthyazol-2-yl)-2.5, diphenyl tetrasodium bromide; Chemicon International, Temecula, CA, USA) dye absorbance as described previously.^9^ MM1.S cells were plated in triplicate at a density of 20 000–30 000 cells per well in 96-well plates (Costar, Cambridge, MA, USA) with media and different concentration of AZD1208 or Dox for 24, 48 and 72 h at 37 °C.

### RNA extraction and quantitative PCR

Total RNA from MM cell lines was isolated and purified with the RNeasy Mini Kit (Qiagen, Hilden, Germany) according to the manufacturer's specifications. cDNA was then synthesized by reverse transcription and then amplified with pairs of gene-specific primers as described previously.^9^ The human quantitative PCR primers used are as follows: *PIM1* F, 5′-GAGTGGATCCGCTACCATCG-3′ and *PIM1* R, 5′-GGCCCCTGATGATCTCTTCG-3′ *PIM2* F, 5′-AGGGATTGAGGATCAGGGGT-3′ and *PIM2* R, 5′-CACAGGTTCTGGGAGGAAGG-3′ *PIM3* F, 5′-GACCCTGACTTTCTCCTGCG-3′ and *PIM3* R, 5′-CCAGCGTTCAAAAGGCACTC-3′ *GAPDH* F, 5′-ACTGTGGATGGCCCCTCCGG-3′ and *GAPDH* R, 5′-GCAGCGCCAGTAGAGGCAG-3′ (Life Technologies).

### Cell cycle analysis and detection of apoptosis

MM cell lines (1 × 10^6^ cells) were cultured for 24 and 48 h in 10% RPMI media alone or with varying concentrations of AZD1208 and harvested and analyzed for apoptosis with an Annexin V/PI Staining Kit (BD Biosciences, San Diego, CA, USA) as described previously.^9^

### Transfection and lentivirus infection

To better assess the role of the PIM kinases in MM cell lines, short hairpin RNA and small interfering RNA (siRNA) was used to knockdown each of the kinases in U266, MM1.S, RPMI and Dox40 cells. Transient transfection-mediated siRNA knockdown was carried out in MM1.S and U266 cells using the Amaxa Cell Nucleofector Kit (Lonza, Basel, Switzerland). On-Target Plus human siRNA for PIM1, PIM2 and PIM3, and On-Target Plus Non-Targeting Pool siRNA was used as a scramble control (Dharmacon, Lafayette, CO, USA). Inducible PIM2 short hairpin RNA knockdowns were cloned in pLKO-Tet-ON plasmids (Addgene, Cambridge, MA, USA) with the following PIM2 primers: F, 5′-CCGGCCAGGATCTCTTTGACTATATCTCGAGATATAGTCAAAGAGATCCTGGTTTTT-3′ and R, 5′-AATTAAAAACCAGGATCTCTTTGACTATATCTCGAGATATAGTCAAAGAGATCCTGG-3′ (Life Technologies). PIM2 short hairpin RNA or pLKO.1 control plasmids were co-transfected with pVSV-G and delta 8.9 plasmid into 293 T cells with FUGENE 6 transfection reagent (Roche, Indianapolis, IN, USA). Virus was then collected at 48/72 h and applied to MM cell lines via spinoculation. Knockdowns were induced with administration of doxycycline at a dose of 100 ng/ml (Selleck Chemicals).

### Statistical analysis

All experiments were carried out a minimum of three times in the laboratory to ensure reliability of the data presented. The viability tests were performed in triplicate for each data point, and the standard deviation was plotted as % error. Additionally, Student's *T*-tests were performed for all graphs to assess the statistical significance of differences, with minimum significance of *P*<0.05. All statistical analysis was carried out on Excel.

## Results

### The PIM kinases are relevant targets in MM

**Figure 1:**
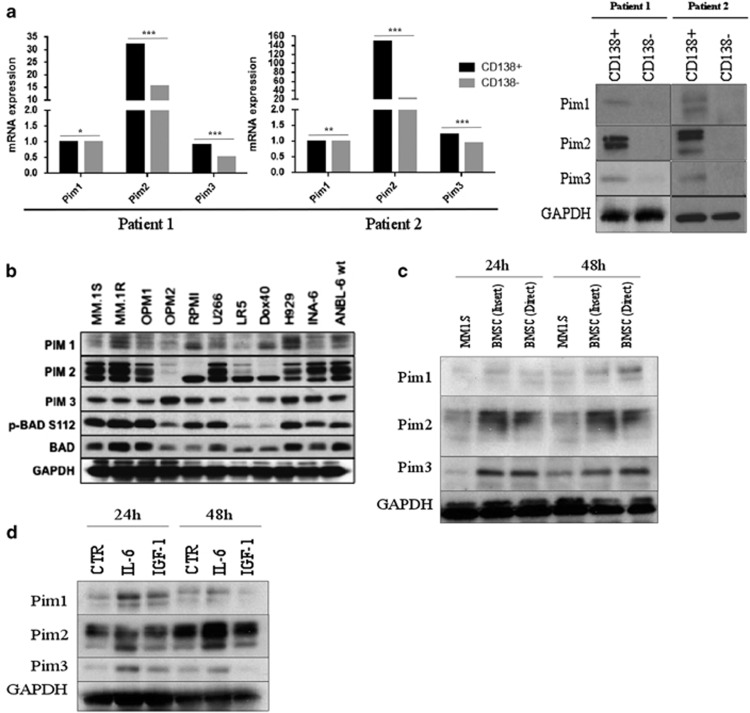
The PIM kinases are relevant targets in MM. (**a**) mRNA and protein expression of Pim1, 2 and 3 in CD138+ and CD138− cells of MM patients were measured. All the three PIM kinases are more highly expressed in CD138+ cells (*P<0.01, **P<0.001, ***P<0.0001, using *t*-test). (**b**) Baseline PIM kinase protein expression was measured via western blotting in human MM cell lines. The three kinases are highly expressed in the majority of MM cell lines tested. (**c**) MM1.S cells were cocultured directly or indirectly via an insert with patient-derived bone marrow stromal cells for 24 and 48 h and blotted for PIM kinase expression. MM1.S cells had a higher expression of the kinases when cocultured with BMSCs. (**d**) MM1.S cells were cultured for 24 and 48 h in the presence of IL-6 (10 ng/μl) and IGF-1 (50 ng/μl) and blotted for Pim kinase expression. Both IL-6 and IGF-1 induced PIM kinase expression.

We first sought to identify whether PIM kinases were a relevant therapeutic target in MM. All three PIM kinases were expressed in patient-derived CD138+ cells. Quantitative PCR and western blot analysis of two different MM patients showed very similar patterns of *PIM* expression: greater *PIM1/2/3* levels in the CD138+ fraction of cells, and a markedly higher expression of *PIM2*, relative to the other two isoforms was noted (Figure 1a). These data have been corroborated by western blot analysis of five additional patient sample sets, and suggests a uniquely important role for PIM2 in MM, relative to PIM1/3. An expression profile of the three kinases in an array of human MM cell lines confirmed that they were expressed in all MM cell lines, with a markedly higher expression of PIM2/3 proteins in a majority of the cell lines, making the kinases applicable to *in vitro* study (Figure 1b). Asano *et al.*^3^ have shown a very strong role for the BMSC compartment in regulating the activation of the PIM kinases in MM. This was confirmed by MM1.S cocultured with patient-derived BMSCs for 24 and 48 h (Figure 1c). PIM kinase levels were upregulated in the context of both direct and indirect contact with the BMSCs, implicating a role of both adhesion and cytokines in mediating this stromal cell/MM cell interaction.^10^ As shown in previous studies, administration of IL-6 and insulin growth factor-1 (IGF-1) over the course of 24 and 48 h was sufficient to elicit a BMSC-like activation of the kinases (Figure 1d).^11^ More specifically, IL-6 was seen to be a more effective mediator, upregulating all three kinases at 24 h and to some extent at 48 h, whereas IGF-1 treatment affected an increase in PIM levels at 24 h, an effect that was diminished at 48 h.

### The PIM kinases are functionally distinct in MM

**Figure 2:**
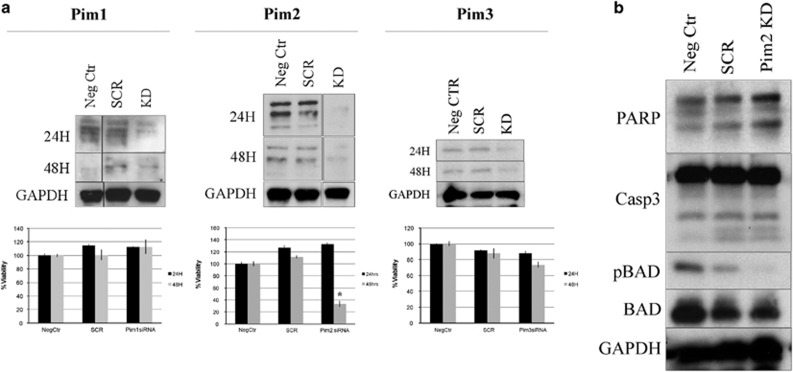
The PIM kinases are functionally distinct. (**a**) Transient knockdown of each of the three kinases was carried out in MM1.S cells and measured via western blotting. MTT viability assays at 24 and 48 h after transfection were performed. The Pim2 knockdown (KD) results in the most significant decrease in cell viability in MM1.S cells at 48 h (*P*=3.23 × 10^−5^, using *t*-test). (**b**) Cells transfected with Pim2 siRNA were also blotted for apoptosis markers at 24 h. Pim2 KD causes PARP and caspase-3 cleavage and commits the cells to apoptosis as early as 24 h. Phosphorylated BAD was also seen to be decreased with the Pim2 KD at 24 h.

Single transient knockdowns of PIM1/2/3 in MM1.S cells via siRNA transfection helped to functionally separate the three isoforms. PIM2 knockdown alone had significant effects on cell survival at 24 and 48 h after transfection, and was seen to be the only isoform implicated in the activation of the apoptotic pathway (Figure 2), whereas PIM 1 and 3 did not result in apoptosis (data not shown). Knockdown of PIM2 in MM1.S cells resulted in the most marked effect on cell viability (seen at 48 h), reaffirming its previously established role in indirectly preventing apoptosis via the phosphorylation of BAD (Figure 2b).^12^ Caspase-3 and PARP cleavage were seen as a result of the PIM2 knockdown as early as 24 h by western blot analysis (Figure 2b).

### PIM2 is involved in the repression of the DDR

**Figure 3:**
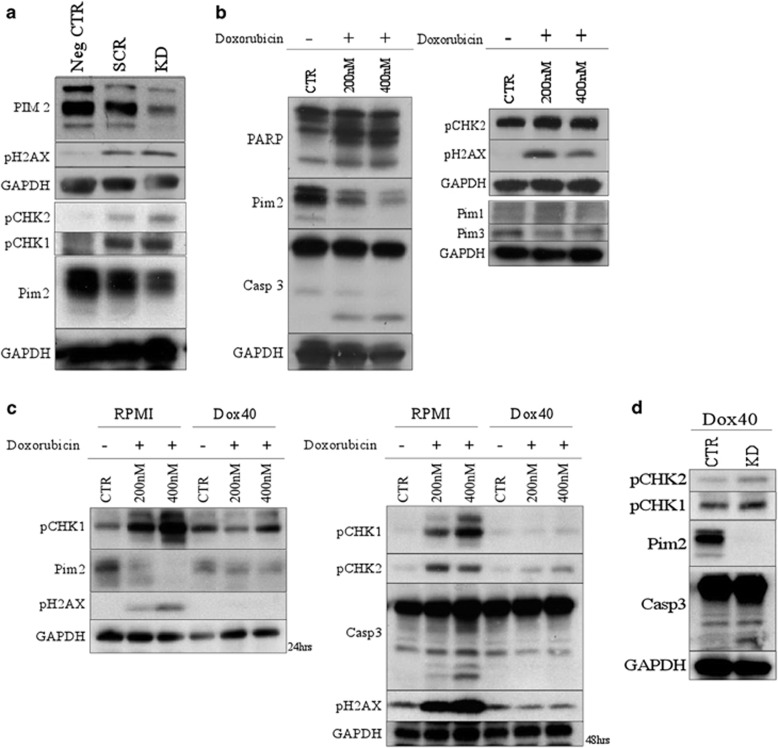
Pim 2 is implicated in the DDR pathway in MM. (**a**) Transient KD of Pim2 in MM1.S cells was performed via transfection of siRNA. Cells were collected at 24 h after transfection and Western blotted for DDR markers. The Pim2 KD results in increased phosphorylation of CHK1, CHK2 and H2AX. (**b**) MM1.S cells were treated with Dox (200 and 400 nm) and harvested at 24 h for western blot analysis. Treatment of MM1.S cells with Dox causes decreased expression of Pim2 and consequently downstream DDR targets at 24 h (while Pim1 and 3 remain unaffected). (**c**) Dox-mediated DNA damage was further analyzed in Dox40 and RPMI cells. The cells were cultured for 24 and 48 h under Dox treatment at 200 and 400 nm, and collected for western blot analysis. Dox treatment of resistant cell line Dox40 does not change Pim2 expression, and downstream targets remain unchanged. (**d**) Pim2 was knocked down in Dox40 cells via short hairpin RNA (shRNA) and the cells were analyzed for the expression of pCHK1, pCHK2 and caspase-3 via western blot analysis. Pim2 knockdown alone was sufficient to activate the DDR pathway in Dox40 cells.

Previous studies have linked the PIM kinases to mediation of the DNA damage response (DDR) pathway in other malignancies.^13^ Single transient knockdown of PIM2 in MM1.S cells caused phosphorylation of several DDR markers including pCHK1, pCHK2, pH2AX and P21, suggesting a role for PIM2 as an upstream regulator of this pathway (Figure 3a).^14^ As a functional control for this analysis of the DNA damage pathway, the effects of Dox treatment on the same markers was analyzed in MM1.S cells. Dox is a DNA-damaging agent that directly binds to double-stranded DNA to trigger the activation of DDR.^15^ Dox administration at the half-maximal inhibitory concentration upregulated pCHK1, pCHK2 and pH2AX as early as 24 h after treatment in MM1.S, mimicking the effects of a PIM2 knockdown (Figure 3b). Interestingly, PIM2 protein levels decreased with Dox treatment, suggesting that PIM2 is directly downstream of the DNA damage site, and acts upstream of ATM/ATR (ataxia telangiectasia-mutated/ATM- and RAD3-related).^16^ This regulatory role further seems to be restricted to PIM2, as PIM1/3 levels remained unchanged in the treated cells.

To determine whether the activation of these DDR markers were dependent on the decrease of PIM2 levels, the pathway was analyzed in Dox-resistant cells. RPMI-8226 and Dox40 cells were treated with Dox and assessed for the activation of the DDR pathway at 24 and 48 h. Interestingly, the pathway was activated in the RPMI cells, which are sensitive to Dox treatment. PIM2 levels were decreased and phosphorylation of the rest of the markers increased. Additionally, the effect on PIM2 levels was seen early in Dox treatment, and the cells did not undergo Caspase-3 cleavage until the 48 h time point, separating the role of Pim2 in this pathway from that of its previously established role in mediating the apoptotic pathway in MM cells. Alternatively, PIM2 levels in the Dox40 cells remained unchanged, along with the rest of the DDR pathway proteins. Single targeted inducible knockdown of PIM2 in the Dox40 cell line was sufficient to affect an increase in phosphorylated DDR markers and bypass the cells' MDR-1-mediated resistance pathway (Figure 3d), as PIM2 activity is downstream of the direct Dox/DNA binding.^17^ These results reaffirmed a dependence on a decrease in PIM2 levels for the activation of downstream DDR markers. Similar tests were performed using γ-irradiation-mediated DNA damage and an increase in PIM2 levels were seen. This is not surprising given the mechanisms of action of γ-irradiation in DDR activation by clustered single/double-stranded breaks,^18^ a mechanism very different from that of Dox-mediated DDR,^19^ and further suggests that PIM2's role in mediating DDR activation is dynamic, depending on the mode of induction (data not shown).

### Exogenous PIM2 activation has differential effects on MM cell survival

**Figure 4:**
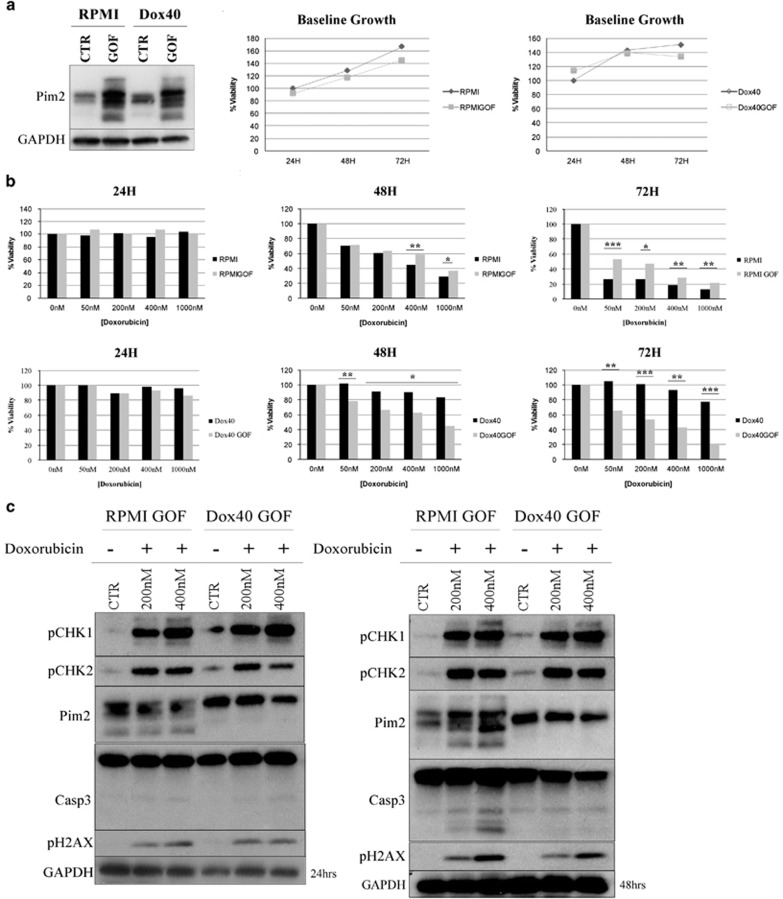
Exogenous expression of Pim2 has differential effects on MM cell survival. (**a**) Pim2 GOF was performed via lentiviral infection of Pim2 knock-in plasmid in RPMI and Dox40 cells and visualized via western blotting at baseline. Viability of GOF lines was assessed via MTT at 24, 48 and 72 h after infection. (**b**) GOF and control cell lines for RPMI and Dox40 were treated with Dox (at 50, 200, 400 and 1000 nm and assessed for viability at 24, 48 and 72 h via MTT. (**c**) RPMI/Dox40 GOF cells were treated with 200 and 400 nm of Dox and blotted for downstream DDR and apoptosis markers at 24 and 48 h after treatment. *P<0.01, **P<0.001 and ***P<0.0001, using *t*-test.

MM cell lines that do not endogenously express high levels of Pim2 were infected with a lentiviral *PIM2* knock-in to further validate its functional importance using a gain-of-function (GOF) model (Figure 4a). The PIM kinases are considered prosurvival factors for MM cells,^3^ and we wanted to confirm this finding in our knock-in system. Cell survival was measured by a series of viability tests. At baseline, knock-in cells did no better than their controls. However, under Dox treatment, there was a slight survival advantage in the knock-in RPMI cells in a dose-dependent manner over the course of 72 h (Figure 4b). To elucidate the functional effect of a *PIM2* knock-in in the Dox-resistant system, the same viability tests were performed in GOF Dox40 cells compared with their control cells (Figure 4b). Interestingly, the same treatment of Dox40 GOF cells reversed their resistance in part to Dox (seen at the 48 h and 72 h time points). The DDR pathway in the knock-in lines was analyzed via western blot analysis at 24 and 48 h under Dox treatment. Phosphorylated CHK1, CHK2 and H2AX were all seen to be upregulated in both RPMI and Dox40 GOF cells (Figure 4c). PIM2 levels were slightly decreased in both cell lines at 24 h upon treatment with 400 nm of Dox.

## Discussion

With each successive study, the PIM kinases have proven to hold a wide variety of functions in regulating MM cancer cell machinery.^20^ The results of this study illuminate a dynamic nature of activity for PIM2, and further support its importance in MM cell survival. The PIM kinases are upregulated in the CD138+ fraction of patient bone marrow, and PIM2 was seen to be the most highly expressed of the three kinases within the cancer compartment. PIM kinases are further overexpressed in MM cell lines, making them an easy subject of *in vitro* study. PIM kinase expression is activated by the presence of BMSC and specifically, by the addition of IL-6 and, to a lesser extent, IGF-1α. Single transient knockdown of the PIM kinases in MM1.S cells pointed to the distinct importance of PIM2 to cell survival. Pim2 transient knockdown further resulted in increased phosphorylation of CHK1, CHK2 and H2AX. Pan-PIM inhibition resulted in activation of pATR, suggesting that PIM2 represses the activation of the DDR pathway via ATR modulation, and may by extension indirectly prevent DDR-mediated apoptosis. Dox treatment of MM1.S cells showed a decrease in PIM2 levels, in addition to activating DDR, suggesting a role for PIM2 directly downstream of DNA damage (Dox binding site). PIM2 knockdown additionally restored the DDR activation in Dox-resistant Dox40 cells, reinforcing its sufficiency for DDR activation.

Overexpression of PIM2, while failing to affect active cell proliferation at baseline, did confer a partial survival advantage to RPMI cells under Dox treatment. This set of studies not only illuminates a potentially new role for PIM2 in mediating the DDR pathway but also may provide an explanation for the inefficacy of current pan-PIM inhibitors in the clinic. PIM2's function in MM is multifaceted and dynamic. Without a thorough understanding of all of its roles, it will be difficult to tailor a drug that can effectively cause cell death without conferring some level of resistance through an alternate pathway.

Ongoing studies aim to understand the basis of interaction between PIM2 and ATM/ATR. It would be beneficial to determine whether PIM2 binds directly to these proteins to affect their inactivation and if in the PIM2 LOF environment there is any conferred functional overlap with PIM1 and PIM3. These studies will contribute to a better understanding of the mode of inhibition of the PIM kinases, allowing the rational design of treatment with this class of drugs in the treatment of MM.
